# The lncRNA MIAT regulates CPT-1a mediated cardiac hypertrophy through m^6^A RNA methylation reading protein Ythdf2

**DOI:** 10.1038/s41420-022-00977-8

**Published:** 2022-04-05

**Authors:** Yiqing Yang, Muisha B. Mbikyo, Junzhe Zhang, Yuan Zhang, Naijin Zhang, Zhao Li

**Affiliations:** Department of Cardiology, The First Hospital of China Medical University, Shenyang, 110001 China

**Keywords:** Heart failure, Methylation

## Abstract

Pathological cardiac hypertrophy is a key contributor in heart failure (HF). Long non-coding RNAs (lncRNAs) and N^6^-methyladenosine (m^6^A) modification play a vital role in cardiac hypertrophy respectively. Nevertheless, the interaction between lncRNA and m^6^A methylase in cardiac hypertrophy is scarcely reported. Here, we constructed a cardiac hypertrophy mouse model by transverse aortic constriction (TAC) surgery and H9c2 cell model by stimulating with AngII. We found that lncRNA MIAT mRNA level, and m^6^A RNA methylation reading protein Ythdf2 mRNA and protein levels, were significantly increased in the cardiac hypertrophy model both in vivo and vitro. MIAT or Ythdf2 overexpression aggravated cardiac hypertrophy, and vice versa. Through bioinformatics prediction, western blotting, FISH, RNA pull-down, and RIP, we found that MIAT bound to Ythdf2 and regulated its expression. Furthermore, we discovered that Ythdf2 function was a downstream of MIAT in cardiac hypertrophy. Finally, we found that MIAT was a necessary regulator of cardiac hypertrophy due to its regulation of the Ythdf2/PPARα/CPT-1a axis. This study indicated a new hypertrophic signaling pathway: MIAT/Ythdf2/PPARα/CPT-1a. The results provided a new understanding of the MIAT and m^6^A RNA methylation reading protein, Ythdf2, function and mechanism in cardiac hypertrophy and highlighted the potential therapeutic benefits in the heart.

## Introduction

Cardiac hypertrophy (CH) is a compensatory functional response to pathological pressure overload, which is characterized by a larger cell surface area, an increased number of sarcomeres, an abnormal thickness of the left ventricular wall, and a decline in cardiac function [1, 2]. With the progress of CH, there is a high occurrence of angina pectoris, arrhythmia, and heart failure [3]. Therefore, research on the pathogenesis of CH has important significance and clinical value for the prevention and treatment of various cardiovascular diseases.

Long non-coding RNAs (lncRNAs) are important epigenetic regulators of gene expression [4]. They are longer than 200 nucleotides and do not code protein directly. However, lncRNAs have complex molecular structures, which can regulate transcription at RNA level, interfere with post-transcriptional modification, and participate in the regulation of signaling pathways and other functions. Previous studies have reported that lncRNAs play a vital role in cancer [5–7]. A number of studies showed that lncRNA participated in cardiac hypertrophy. For example, Cardiac Hypertrophy-Associated Epigenetic Regulator (Chaer) interacted with polycomb repressor complex 2 (PRC2) and suppressed the histone H3 lysine 27 methylation, thus led to cardiac hypertrophy [8]. Cardiac Hypertrophy-Associated Transcript (Chast) was upregulated in both mouse TAC model and aortic stenosis patients [9]. Myocardial Infarction–Associated Transcript (MIAT) inhibited miR-150 expression and aggravated cardiac hypertrophy in AngII-induced cells [10]. However, the precise role of MIAT in CH is still unclear.

N^6^-methyladenosine (m^6^A) modification is the most abundant RNA modification in eukaryocytes [11]. There are three key enzymes during this dynamic modification: methyltransferase (writers), demethylase (erasers), and methylation reading protein (readers). Recent studies have shown that m^6^A RNA modification was related to cardiac remodeling. For instance, a writer named methyltransferase 3 (METTL3) increased, and an eraser called FTO alpha-ketoglutarate dependent dioxygenase (FTO) decreased, in human heart failure samples [12]. By interfering with the expression level of m^6^A methylase with various techniques, researchers found that the cardiac m^6^A RNA modifications could regulate gene expression and influenced cardiac hypertrophy [13]. YTH N6-methyladenosine RNA-binding protein 2 (Ythdf2) is the first reader to be found. It speeds up the degradation of transcripts modified by m^6^A in the way of enlisting the CCR4-NOT deadenylase complex directly [14]. It is unclear whether Ythdf2 is presented in cardiovascular diseases and affects cardiac hypertrophy.

Peroxisome proliferator-activated receptorα (PPARα) signaling is an important pathway which functions in many cellular metabolic processes including cardiovascular diseases [15]. There are three isoforms: PPARα, PPARβ/δ and PPARγ [16]. PPARα is mainly expressed in the heart, liver, kidney, and brown fat tissue. It regulates energy homeostasis by activation of fatty acid catabolism and stimulation of gluconeogenesis [17]. Carnitine palmitoyl transferase-1a (CPT-1a) is a key component of this pathway and acts as rate-limiting enzyme in mitochondrial fatty acid oxidation [18]. The important role of PPARα/CPT-1a in CH has been studied [19, 20]. However, whether this signaling is also involved in MIAT regulated CH progression remains unknown.

In this study, we demonstrated that MIAT influenced AngII-induced cardiac hypertrophy by regulating the expression of a key m^6^A RNA methylation enzyme, Ythdf2, and may influence its downstream target genes PPARα/CPT-1a.

## Materials and methods

### Animal model

Healthy male mice (age, 8 weeks; weight,19–21 g) on a C57BL/6 J background were purchased from the Laboratory Animal Department of China Medical University. The mice were housed in a room with uniform temperature (20–24 °C) and humidity (50–60%) under a 12-h light–dark cycle. The mice were randomly divided into two groups, *n* = 8, respectively. They were kept on a standard diet for at least one week before the experiment. The cardiac hypertrophy pressure overload animal model was produced by transverse aortic constriction (TAC) as described previously [21]. Sham operations group were treated similarly but aortic constriction was not performed. This study conformed to the Guide for the Care and Use of Laboratory Animals which was published by the National Institutes of Health (NIH Publications No.8023, revised 1996). All protocols were approved by the Animal Experimentation Ethical Standards Committee of China Medical University (SCXK-2013–0001; Shenyang; China).

### Echocardiography

Transthoracic echocardiography was performed on the mice under isoflurane anesthesia (1% isoflurane; 0.6-liter flow of O_2_). The condition of the heart was visualized by Vevo 2100 VisualSonics system (FUJIFILM VisualSonics Inc., Toronto, Canada) under B- and M-mode images. LVPWd indicated left ventricular posterior wall dimensions at diastole and IVSd indicated interventricular septal thickness at diastole.

### Hematoxylin-eosin staining and measurement of cell area

Mice heart tissue was taken and placed in a 10% formalin fixation solution for 24 h, then the water in the tissue was gradually removed through low concentration to high concentration alcohol. The tissue was placed in xylene for transparency and then embedded in paraffin wax in order to cut it into thin slices (usually 5 microns). Paraffin in tissue was removed with xylene, followed by high to low concentrations of alcohol (100%, 95%, 80%), and finally distilled water. The slice was placed in hematoxylin for 4 min and rinsed in eosin for 2 min. Finally, they were dehydrated with 100% alcohol. The slides were then air-dried, dripped with Canada gum and sealed with cover glass.

The images were visualized by Leica microscope (DM600, Leica Microsystems GmbH, Germany) under x10 and x40 magnifications. The cross-sectional area of cardiomyocyte was evaluated by ImageJ (Ver 1.8.0).

### Cell culture and treatment

The rat heart-derived H9c2 cell line was purchased from the Chinese Academy of Sciences (Shanghai, China) and cultured in Dulbecco’s modified Eagle medium (DMEM) with 10% FBS (HyClone). All cells were grown in a 5% CO_2_, 37 °C incubator. To establish the cardiac hypertrophy model, H9c2 cells were starved with DMEM without FBS for 24 h before medicated with 0.2 uM Angiotensin II (AngII) for 48 h.

### Antibodies and reagents

The antibodies were used as follows: Anti-ANP antibody (ab225844, abcam, WB:1:1000), Anti-BNP antibody (ab19645, abcam, WB:1:500), Anti-β-MHC antibody (ab172967, abcam, WB:1:1000), Anti-Ythdf2 antibody (24744–1-AP, proteintech, IF:1:200; WB:1:1000), Anti-CPT-1a (66039-1-Ig, proteintech, IF:1:200, WB:1:1000), Anti-PPARα (AF5301, Affinity, WB:1:1000) GAPDH antibody (abs132004, absin, WB:1:5000), anti-β-tubulin antibody (10094-1-AP, Proteintech, WB:1:1000), goat anti-rabbit IgG-HRP (abs20002, absin, WB:1:10000), goat anti-mouse IgG-HRP (abs20001, absin, WB:1:10000). AngII (HY-13948) was purchased from MCE (China).

### RNA interference and gene overexpression

For RNA interference, MIAT siRNA (siMIAT), Ythdf2 siRNA (siYthdf2), and scramble siRNA (negative control) were designed and obtained from RIBOBIO Co., Ltd. (Guangzhou, China). MIAT and Ythdf2 silencing was performed with jetPRIME transfection reagent (PolyPlus, France) when transfecting cells at 50% confluency according to the manufacturer’s instructions. Three sequences were provided to avoid off-target effects:

MIAT siRNA-1: GCTTCACAACCCAGGCTTA

MIAT siRNA-2: GCTAACCTCTGGCTCCTTT

MIAT siRNA-3: GTTGGGTATTTACTCTTCA

Ythdf2 siRNA-1: GGGATTGACTTCTCAGCAT

Ythdf2 siRNA-2: GGGCTGATATTGCTAGCAA

Ythdf2 siRNA-3: GGTTCTGGATCTACTCCTT

For gene overexpression, pcDNA-MIAT (MIAT) and pcDNA empty vector, pcDNA-Ythdf2 (Ythdf2), and pcDNA empty vector were designed and purchased from GeneChem Co., Ltd. (Shanghai, China). Transfection was performed in H9c2 cell with Lipofectamine 3000 (Invitrogen, USA) before stimulation with 0.2 uM AngII for 48 h.

### Fluorescence in situ hybridization and immunofluorescence

Cy3-labeled MIAT probes were designed and synthesized by RIBOBIO Co., Ltd. (Guangzhou, China). H9c2 was fixed in 4% paraformaldehyde and hybridized with hybridization buffer which contained MIAT probes in the dark place overnight at 37 °C. The following day, primary antibody Ythdf2 (1:200) and CPT-1a (1:200) were incubated at 37 °C overnight. FITC-labeled Goat anti-rabbit IgG-HRP (1:200) and Alexa Fluor 633-labeled Goat anti-mouse IgG-HRP (1:200) were incubated at 37 °C for 1 h. The nuclei were stained with DAPI (Solaribo, Beijing, China). The confocal microscope was used to visualize the slides (ZEISS LSM 900 with Airyscan 2, Germany). The images were processed with ZEN microscopy software.

### RNA pull-down assay

The MIAT probes end-labeled with desthiobiotin were designed and synthesized by RIBOBIO Co., Ltd. (Guangzhou, China). RNA pull-down assay was performed according to the manufacturer’s instructions (GENESEED, P0201). Briefly, streptavidin magnetic beads were pre-washed, and cell lysate was prepared. Labeled MIAT was binding to beads with incubation for 30 min at room temperature with agitation. A master mix containing lysate could bind RNA-Binding Protein to RNA with incubation period of 60 min at 4 °C with rotation. Then, RNA-Binding Protein complexes were washed and eluted. The samples were boiled at 95–100 °C for 10 min and then electrophoresed for western blot analysis.

### RNA Immunoprecipitation (RIP)

H9c2 cells in a 10-cm dish at 75% confluency were harvested and lysed. RNA Immunoprecipitation was performed according to the manufacturer’s instructions of RNA Immunoprecipitation Kit (GENESEED, P0101). Briefly, magnetic beads were preprocessed and connected with RIP antibody, and then added with cells lysis buffer and rotated for 2 h at 10 r/min. Buffer B was used to wash magnetic-beads complex. RNAs of Input and IP groups were extracted with RC Columns and analyzed by RT-qPCR.

### Chromatin immunoprecipitation-PCR (ChIP-PCR)

The protocol was referred to the previous article [22]. Briefly, the primers of Lnc-MIAT which covered the predicted binding sites of Ythdf2 were designed. Use formaldehyde to cross-link target protein and DNA, and fragment chromatin by sonication. The protein-DNA complex was immunoprecipitated by specific antibody. Then the purified DNA fragments were analyzed by PCR.

### RT-qPCR

Total RNA was extracted from H9c2 using Trizol reagent (Invitrogen). Reverse transcription was performed by the PrimeScript RT reagent Kit with gDNA Eraser (Takara, RR047A). TB Green Premix Ex Taq II (Takara, RR820A) was used in amplification. qPCR was performed using Applied Biosystems 7500 Fast Real-Time PCR System, and the average cycle thresholds (Ct) values were recorded in this analysis system. The mRNA expression was calculated using 2^−△△Ct^ method after introducing GAPDH as endogenous control for calibration. The primers designed from Sangon Biotech (Shanghai) Co., Ltd are listed in Table 1.

**Table 1:** Sequences of polymerase chain reaction primers.

Target ID	Sequence
ANP	Forward: 5'-GAGCGAGCAGACCGATGAAGC-3'
	Reverse: 5'-TCCATCTCTCTGAGACGGGTTGAC-3'
BNP	Forward: 5'-AGTCTCCAGAACAATCCACGATGC-3'
	Reverse: 5'-GCCTTGGTCCTTTGAGAGCTGTC-3'
β-MHC	Forward: 5'-CCAGAACACCAGCCTCATCAACC-3'
	Reverse: 5'-CACCGCCTCCTCCACCTCTG-3'
YTHDF2	Forward: 5'-TTGCCTCCACCTCCACCACAG-3'
	Reverse: 5'-CCCATTATGACCGAACCCACTGC-3'
MIAT	Forward: 5'-GTGCCTTTCTGGTCTGTTCCTTCC-3'
	Reverse: 5'-CCGCCATCCAAGCCGTTAGTG-3'
GDPAH	Forward: 5'-CCGCCATCCAAGCCGTTAGTG-3'
	Reverse: 5'-GTGGTGCAGGATGCATTGCTCTGA-3'

### Co-immunoprecipitation and western blot assay

Briefly, the H9c2s were collected using lysate buffer which added protease inhibitors (Roche, Switzerland) on ice. The cell lysates were incubated with specific antibodies and 35 ul of Protein A/G beads at 4 °C for 12 h. The bound complexes were washed with cell lysis three times and subjected to 8% or 12% SDS-polyacrylamide gel electrophoresis according to molecular weights and transferred to PVDF membranes provided from Millipore (USA). After transferring, the membranes were blocked in Trisbuffered saline containing Tween (TBST) with 5% bovine serum albumin (BSA) at room temperature for an hour. Primary antibodies were incubated at 4 °C rotationally at the lowest speed overnight. After 12–16 h, the membranes were washed in TBST for 45 min. Next, the second incubation was conducted using goat anti-rabbit IgG-HRP at room temperature for 60 min and washed with TBST for another 45 min. Finally, the membranes were soaked in SuperSignal^TM^ ECL substrate (Thermo Scientific) and captured using enhanced chemiluminescence imager.

### Phalloidin staining and measurement of cell area

H9c2s were fixed in 4% paraformaldehyde (room temperature) for 15 min, washed with PBS 3 times, permeabilized with 0.5% Triton X-100 in PBS for 15 min. The H9c2 were stained with phalloidin (Yeasen, Shanghai, China) and the nuclei were stained with DAPI (Solaribo, Beijing, China). The Olympus fluorescence microscope was used to visualize the slides at x400 magnification (AX70, Olympus Corporation, Tokyo, Japan). The views of cells were selected at random. The relative cell surface area was processed with ImageJ (Ver 1.8.0).

### RNA stability analysis

Cells were treated by actinomycin D for 0, 4, 8, 12 h. RNA samples were collected at each time. RT-qPCR was used to measure the stability level of CPT-1a mRNA.

### Statistical analysis

For each set of experiments, the sample size was chosen to ensure adequate power to detect variations. Data were calculated as mean ± standard deviation (SD) and analyzed with two-tailed unpaired *T*-test, one-way ANOVA, or two-way ANOVA followed by Tukey’s multiple comparisons test. All data were processed by SPSS 24.0 and Graphpad Prism 8.0 software. *P* < 0.05 was regarded as a statistically significant difference.

## Results

### MIAT and Ythdf2 expression levels are associated with cardiac hypertrophy in vivo and vitro

**Fig. 1:**
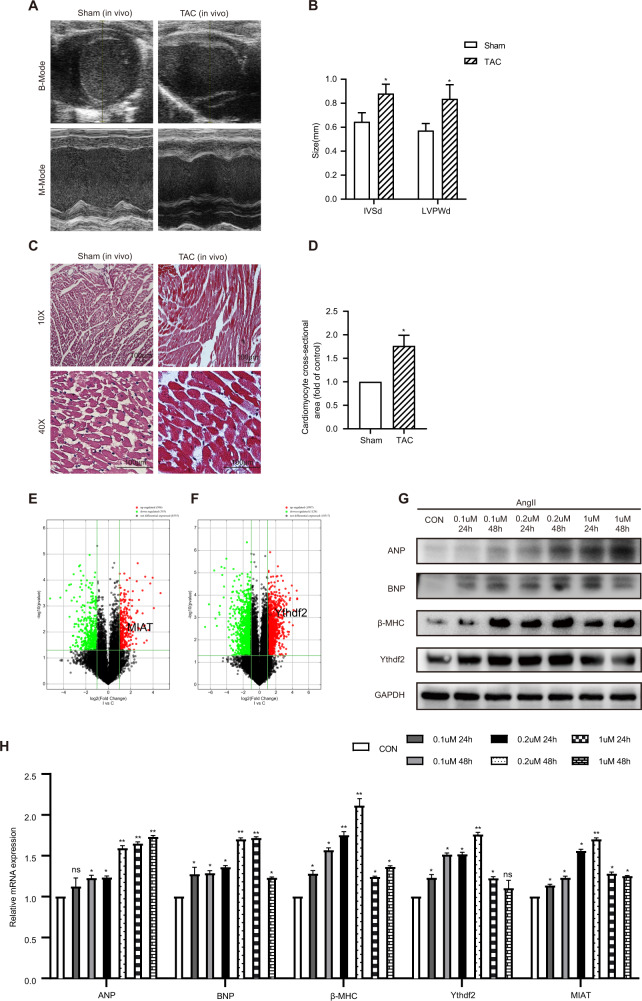
MIAT and Ythdf2 expression levels were associated with cardiac hypertrophy in vivo and in vitro. **A** Echocardiography of the Sham and TAC groups in B- and M-modes. **B** IVSd and LVPWd of the Sham (*n* = 8) and TAC groups (*n* = 8) measured by M-mode echocardiography. **C** H&E staining for x10 and x40 magnifications. Scale bar:100 um. **D** The relative cardiomyocytes cross-sectional area. **E** Volcano Plot of the lncRNA of Sham and TAC groups. **F** Volcano Plot of the mRNA of Sham and TAC groups. **G** Western blot of the expression levels of ANP, BNP, β-MHC, and Ythdf2 at 24 h or 48 h for different concentration gradient. **H** Relative mRNA expression levels of ANP, BNP, β-MHC, Ythdf2, and MIAT at 24 h or 48 h for different concentration gradient detected by RT-qPCR. TAC transverse aortic constriction, LVPWd left ventricular posterior wall thickness at end-diastole, IVSd interventricular septal at end-diastole. All data were expressed as mean ± SD (*n* = 3). ^∗∗∗^*p* < 0.001, ^∗∗^*p* < 0.01, and ^∗^*p* < 0.05 vs control group, ns meant no significance.

We used echocardiography and H&E staining to demonstrate that cardiac hypertrophy was successfully induced by TAC. First, we used echocardiography to visualize the cardiac parameters under B- and M-modes. The IVSd and LVPWd in TAC mice were increased significantly than in Sham group mice (Fig. 1A, B). In addition, by H&E staining, histological analysis of cardiomyocytes of TAC group and Sham group the for x10 and x40 magnifications (Fig. 1C) was obtained. The cardiomyocyte cross-sectional area significantly increased in the TAC group compared with the Sham group (Fig. 1D). Subsequently, the mice were sacrificed and hearts were harvested. We used TRIzol reagent (Invitrogen) for RNA extraction and then sent the samples to make Mouse LncRNA Array V4.0 (Arraystar, USA) to profile lncRNA and mRNA expression. We searched all the m^6^A methyltransferase (writers), demethylase (erasers), and methylation reading protein (readers) in the differentially Expressed mRNAs result. Then we found that the Ythdf2 (readers) was highly expressed and there was a significant difference in cardiac hypertrophy model (logFC = 1.114; *P* = 0.00057) (Fig. 1F). The result showed that the expression of lncRNAs were significantly different in cardiac hypertrophy model and control group by high-throughput microarray detection (GSE169580), among which lnc-MIAT was significantly increased (Fig. 1E).

We then established cardiac hypertrophy model in H9c2 cells by using AngII at 0.1 uM, 0.2 uM, and 1 uM for 24 h or 48 h, respectively. Successful establishment of the cardiac hypertrophy model was confirmed by an obviously increased protein expression level of ANP, BNP, β-MHC and Ythdf2 in western blot (Fig. 1G) and a clearly increased relative mRNA expression level of ANP, BNP, β-MHC, Ythdf2, and MIAT in RT-qPCR (Fig. 1H). We chose 0.2uM AngII stimulated for 48 h as the best according to western blot and RT-qPCR analysis.

### MIAT mediated AngII-induced hypertrophy

**Fig. 2:**
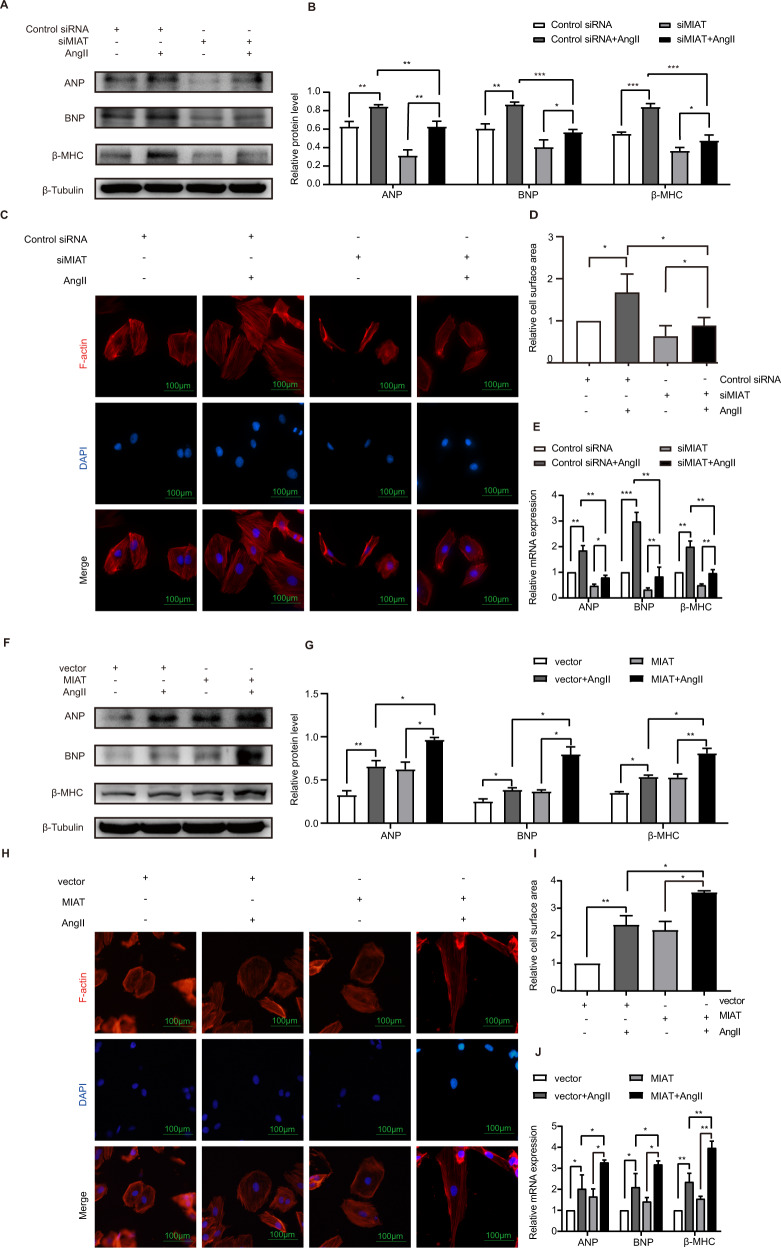
MIAT mediated AngII-induced hypertrophy. H9c2 cells transfected with control siRNA or MIAT siRNA (**A**–**E**). **A, B** Western blot and the relative protein levels of ANP, BNP, and β-MHC. **C** Phalloidin staining. F-actin were stained with fluorescent phalloidin (red), and the nuclei were stained with DAPI (blue). Scale bar: 100 um. **D** Relative cell surface area measured by phalloidin staining. **E** RT-qPCR of the relative mRNA expression levels of ANP, BNP, and β-MHC. H9c2 cells transfected with vector empty or MIAT plasmid (**F**–**J**). **F, G** Western blot analysis and the relative protein level of ANP, BNP, and β-MHC. **H** Phalloidin staining. F-actin in the H9c2 cells were stained with fluorescent phalloidin (red), and the nuclei were stained with DAPI (blue). Scale bar: 100 um. **I** Relative cell surface area measured by phalloidin staining. **J** RT-qPCR of the relative mRNA expression levels of ANP, BNP, and β-MHC. All data were expressed as mean ± SD (*n* = 3). ^∗∗∗^*p* < 0.001; ^∗∗^*p* < 0.01; ∗*p* < 0.05.

The knockdown efficiency of siMIAT was identified by downregulation of MIAT mRNA expression (Supplemental Fig. 1A). Western blot analysis (Fig. 2A) and quantification of the relative protein level (Fig. 2B) showed that AngII promoted the expression of ANP, BNP, and β-MHC in control siRNA group. Following the treatment of AngII, siMIAT decreased the levels of ANP, BNP, and β-MHC relative to control siRNA group. These results showed that MIAT promoted the cardiac hypertrophy phenotype of AngII-treated H9c2 cells. To further confirm the promoting role of MIAT in H9c2 cardiac hypertrophy phenotype, phalloidin staining showed that AngII increased the cell surface area in H9c2 while MIAT knockdown significantly inhibited cardiac hypertrophy following AngII treatment (Fig. 2C, D). RT-qPCR assay indicated that the promotive effect of AngII on cardiac hypertrophy phenotype was suppressed by MIAT knockdown (Fig. 2E).

Contrary to effects observed in the H9c2 cells after the knockdown of MIAT, the overexpression of MIAT significantly increased the cardiac hypertrophy phenotype of AngII-treated cells. AngII increased the expression of ANP, BNP, and β-MHC in empty plasmid transfected group. Following the AngII treatment, MIAT overexpression increased the levels of ANP, BNP, and β-MHC protein expression relative to the empty vector group (Fig. 2F, G). In line with these findings, phalloidin staining revealed that AngII significantly increased cell surface area, and MIAT overexpression further enhanced the cell surface area of the AngII-treated H9c2s (Fig. 2H, I). RT-qPCR assay demonstrated that stimulative effect of AngII on H9c2 cardiac hypertrophy phenotype was enhanced by MIAT overexpression (Fig. 2J).

### Ythdf2 participated in AngII-induced hypertrophy

**Fig. 3:**
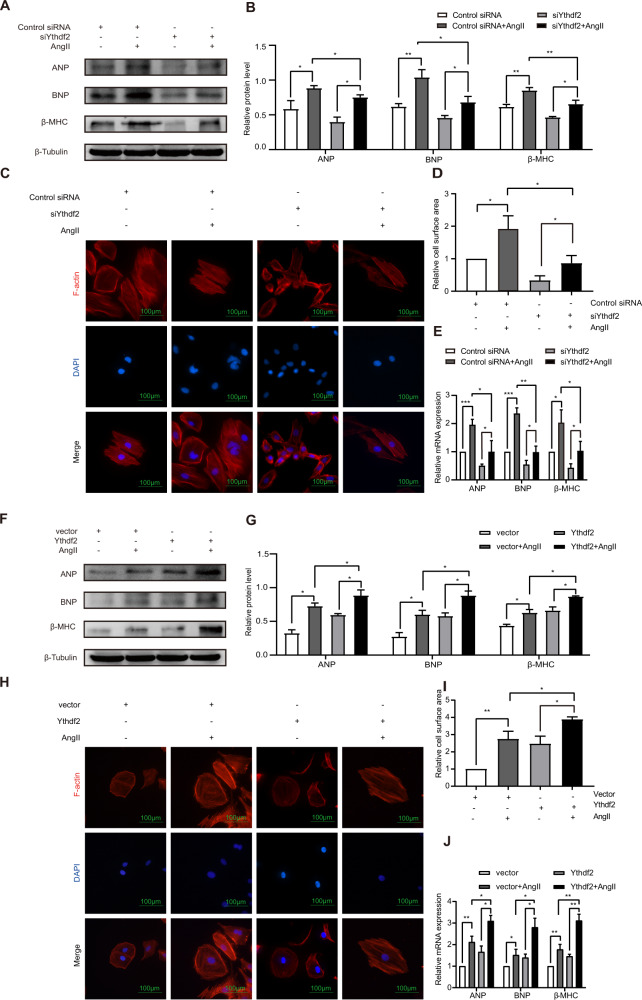
Ythdf2 participated in AngII-induced hypertrophy. H9c2 cells transfected with control siYthdf2 or Ythdf2 siRNA (**A**–**E**). **A, B** Western blot and the relative protein levels of ANP, BNP, and β-MHC. **C** Phalloidin staining. F-actin were stained with fluorescent phalloidin (red), and the nuclei were stained with DAPI (blue). Scale bar: 100 um. **D** Relative cell surface area measured by phalloidin staining. **E** RT-qPCR of the relative mRNA expression levels of ANP, BNP, and β-MHC. H9c2 cells transfected with vector empty or Ythdf2 plasmid (**F**–**J**). **F, G** Western blot analysis and the relative protein levels of ANP, BNP, and β-MHC. **H** Phalloidin staining. F-actin in the H9c2 cells were stained with fluorescent phalloidin (red), and the nuclei were stained with DAPI (blue). Scale bar: 100 um. **I** Relative cell surface area measured by phalloidin staining. **J** RT-qPCR of the relative mRNA expression levels of ANP, BNP, and β-MHC. All data were expressed as mean ± SD (*n* = 3). ^∗∗∗^*p* < 0.001, ^∗∗^*p* < 0.01, and ^∗^*p* < 0.05.

The knockdown efficiency of siYthdf2 was identified by downregulation of Ythdf2 mRNA expression (Supplemental Fig. 1B). Western blot analysis (Fig. 3A) and quantification of the relative protein level (Fig. 3B) revealed that AngII promoted the expression of ANP, BNP, and β-MHC in control siRNA group. Following the treatment of AngII, siYthdf2 decreased the levels of ANP, BNP, and β-MHC relative to control siRNA group. These results demonstrated that Ythdf2 promoted the cardiac hypertrophy phenotype of AngII-treated H9c2 cells. To further confirm the promoting role of Ythdf2 in H9c2 cardiac hypertrophy phenotype, phalloidin staining showed that AngII increased the cell surface area in H9c2 while Ythdf2 knockdown significantly inhibited cardiac hypertrophy following AngII treatment (Fig. 3C, D). RT-qPCR assay showed that the promotive effect of AngII on cardiac hypertrophy phenotype was suppressed by Ythdf2 knockdown (Fig. 3E).

Contrary to effects observed in the H9c2 cells after the knockdown of Ythdf2, the overexpression of Ythdf2 significantly increased the cardiac hypertrophy phenotype of AngII-treated cells. AngII increased the expression of ANP, BNP, and β-MHC in empty plasmid transfected group. Following the AngII treatment, Ythdf2 overexpression increased the levels of ANP, BNP, and β-MHC protein expression relative to the empty vector group (Fig. 3F, G). Consistent with these findings, phalloidin staining showed that AngII significantly increased cell surface area and Ythdf2 overexpression further enhanced the cell surface area of the AngII-treated H9c2s (Fig. 3H, I). RT-qPCR assay revealed that the promotive role of AngII on H9c2 cardiac hypertrophy phenotype was enhanced by Ythdf2 overexpression (Fig. 3J).

### The interaction between MIAT, Ythdf2, and CPT-1a

**Fig. 4:**
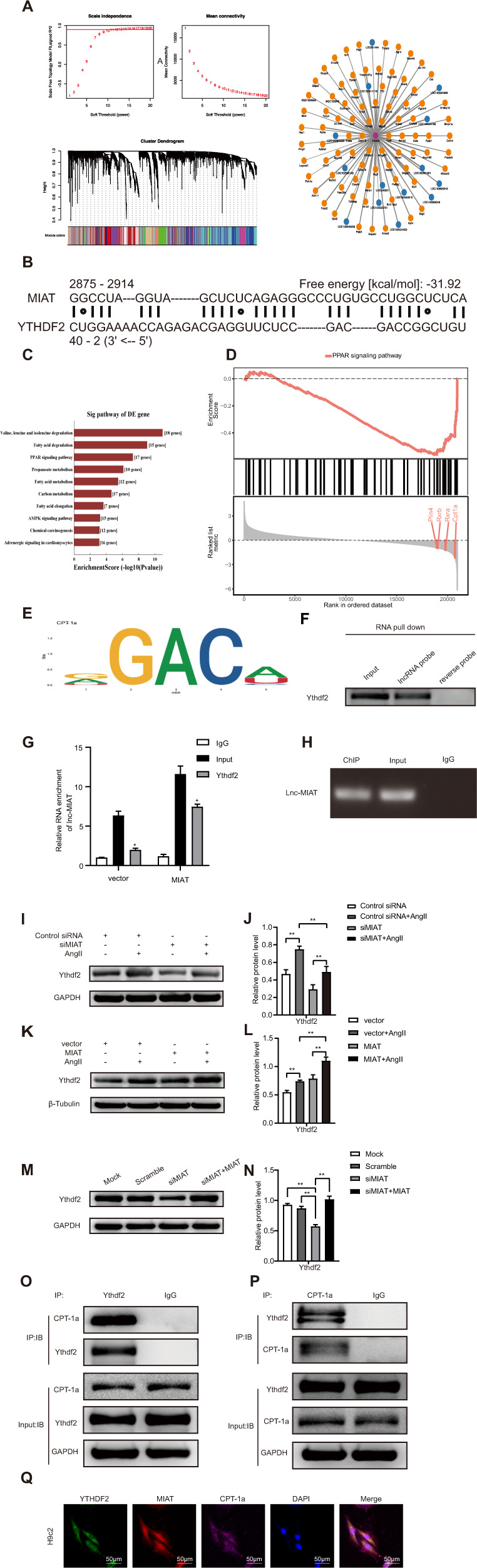
The interaction between MIAT, Ythdf2, and CPT-1a. **A** WGCNA predicted the interaction between MIAT and YTHDF2 (weight = 0.438). **B** RI search predicted the binding sites of MIAT and Ythdf2. **C** Differentially expressed lncRNAs were mainly involved in signal pathways such as PPAR and AMPK. **D** Possible genes that may be regulated by Ythdf2 in the PPAR signal pathway. **E** MEME analysis suggested the presence of m^6^A motif on CPT-1a. **F** Immunoblotting for specific associations of Ythdf2 with MIAT from RNA pull-down assays. **G** RIP assays were performed using antibody against Ythdf2. MIAT expression levels were measured by RT-qPCR. **H** Verification of predicted Ythdf2 binding sites by ChIP-PCR. **I, J** Western blot analysis and the relative protein level of Ythdf2 in AngII-treated H9c2 cells transfected with control siRNA or siMIAT. **K, L** Western blot analysis and quantification of the relative protein level of Ythdf2 in AngII-treated H9c2 cells transfected with vector empty or MIAT plasmid. **M, N** Western blot analysis and the relative protein level of Ythdf2 in H9c2 cells transfected with mock, scramble, siMIAT, siMIAT+MIAT. **O, P** Co-immunoprecipitation analysis of the endogenous interaction between Ythdf2 and CPT-1a. **Q** RNA-FISH and immunofluorescence staining indicated the colocalization of MIAT (red), Ythdf2 (green), and CPT-1a (purple) in H9c2 cells. Scale bar: 50 μm. All data were presented as mean ± SD (*n* = 3). ^∗∗∗^*p* < 0.001; ^∗∗^*p* < 0.01; ^∗^*p* < 0.05.

To explore the mechanism of MIAT in cardiac hypertrophy, we first investigated whether MIAT and Ythdf2 actually interacted. We analyzed the microarray data and used bioinformatics, and found that there was a co-expression relationship between MIAT and Ythdf2. Data analysis of Ythdf2-regulated lncRNAs by weighted gene co-expression network analysis (WGCNA) revealed an interaction between Ythdf2 and MIAT (weight = 0.438) (Fig. 4A). RI search predicted the trans regulation between MIAT and Ythdf2 and revealed their binding sites (Fig. 4B). Differentially expressed lncRNAs were found to be mainly involved in PPAR and AMPK signaling pathways through GO and Pathway analysis (Fig. 4C). Previous studies have shown that cardiac hypertrophy is closely related to the PPARα pathway [23–25], so we chose the PPARα signaling pathway as a candidate. WGCNA analysis indicated that CPT-1a, PLIN4, RXRB, and RXRA were possible target genes for RNA methylation key enzyme, Ythdf2, in the pathway (Fig. 4D). Carnitine palmitoyl transferase-1a (CPT-1a) is a key enzyme in mitochondrial fatty acid oxidation and has been reported in cardiac hypertrophy via PPAR/CPT-1a signaling pathway [26–28]. Multiple Em for Motif Elicitation (MEME) analysis revealed the presence of m^6^A motif on CPT-1a (Fig. 4E). We worked on the assumption that Ythdf2, as an m^6^A methylation reading protein, may bind to the m^6^A site of CPT-1a to promote CPT-1a mRNA degradation. Immunoblotting independently confirmed that Ythdf2 interacted directly with MIAT (Fig. 4F). Additionally, RIP assays also showed that MIAT was significantly enriched in pull-downs with antibody against Ythdf2 both in the vector group and MIAT overexpression group, as determined by measuring coprecipitated RNA by RT-qPCR (Fig. 4G). The identified Ythdf2 binding sites were confirmed by ChIP-PCR using Ythdf2 antibody (Fig. 4H). Moreover, western blot analysis and quantification of the relative protein level showed that, under the stimulation of AngII, Ythdf2 was decreased in H9c2 cells transfected with siMIAT compared to control group (Fig. 4I, J), whereas overexpression of MIAT increased the Ythdf2 protein level in AngII-treated H9c2 cells (Fig. 4K, L). We also found that Ythdf2 levels significantly decreased in H9c2 cells with silenced MIAT, while notably rescued after the re-overexpression of MIAT (Fig. 4M, N). These results indicated that Ythdf2 specifically interacted with MIAT in H9c2 cells.

To explore the mechanism of Ythdf2 in cardiac hypertrophy, we investigated whether Ythdf2 and CPT-1a actually interacted, which may affect the cardiac hypertrophy in H9c2 cells. The result of endogenous co-immunoprecipitation indicated that CPT-1a was a novel protein which bound with Ythdf2 (Fig. 4O, P). Moreover, RNA-FISH and immunofluorescence staining assay were performed to show the colocalization of MIAT, Ythdf2, and CPT-1a. It revealed enriched signals of MIAT-Ythdf2-CPT-1a complex in the cytoplasm of H9c2 cells. Ythdf2 and CPT-1a protein was distributed in the cytoplasm, whereas MIAT was distributed in the nucleus and cytoplasm (Fig. 4Q).

### Ythdf2 and CPT-1a function as downstream of MIAT in cardiac hypertrophy

**Fig. 5:**
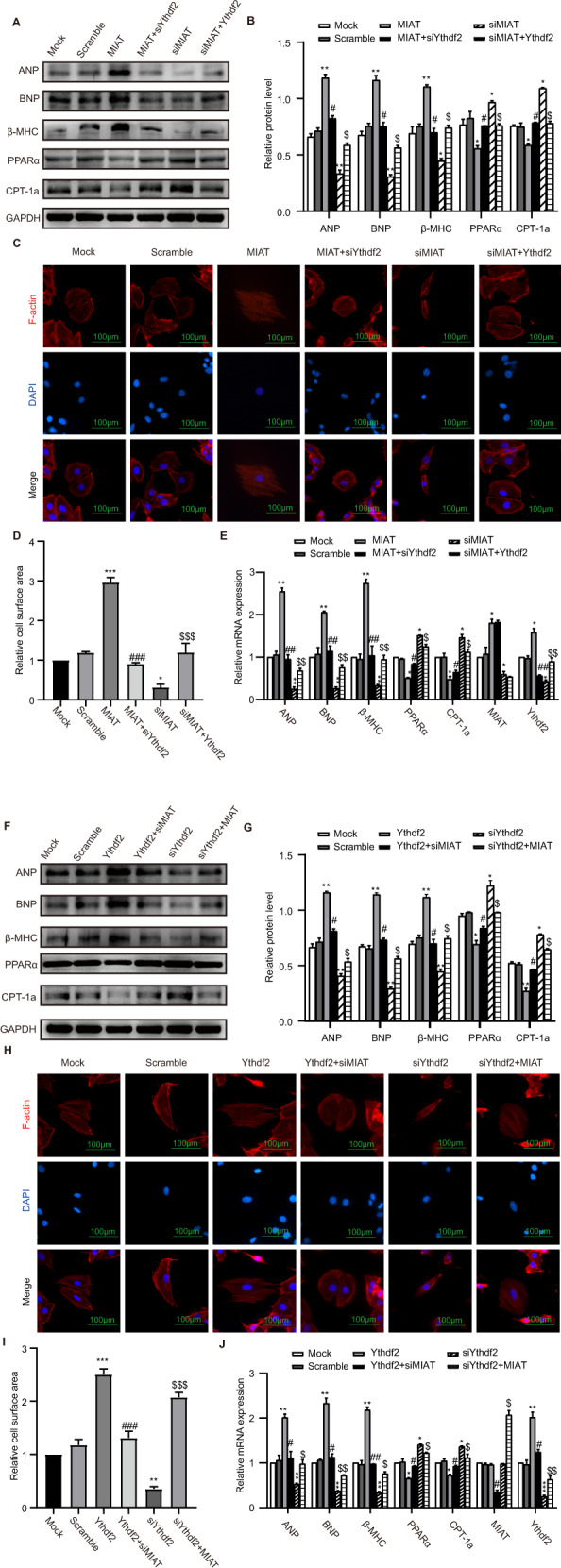
Ythdf2 and CPT-1a function as downstream of MIAT in cardiac hypertrophy. H9c2 cells transfected with MIAT, MIAT + siYthdf2, siMIAT, siMIAT+Ythdf2 (**A**–**E**). **A, B** Western blot analysis and relative protein levels of ANP, BNP, β-MHC, PPARα and CPT-1a. **C, D** Phalloidin staining and the relative cell surface area: F-actin were stained with fluorescent phalloidin (red) and the nuclei were stained with DAPI (blue). Scale bar: 100 um. **E** RT-qPCR of the relative mRNA expression levels of ANP, BNP, and β-MHC, PPARα, CPT-1a, MIAT and Ythdf2. H9c2 cells transfected with Ythdf2, Ythdf2+siMIAT, siYthdf2, siYthdf2+MIAT (**F**–**J**). **F, G** Western blot analysis and relative protein levels of ANP, BNP, and β-MHC, PPARα and CPT-1a. **H, I** Phalloidin staining and the relative cell surface area: F-actin were stained with fluorescent phalloidin (red) and the nuclei were stained with DAPI (blue). Scale bar: 100 um. **J** RT-qPCR of the relative mRNA expression levels of ANP, BNP, β-MHC, PPARα, CPT-1a, MIAT and Ythdf2. ^∗∗∗^*p* < 0.001 and ^∗∗^*p* < 0.01 and ^∗^*p* < 0.05 vs control group. ^###^*p* < 0.001 and ^##^*p* < 0.01 and ^#^*p* < 0.05 vs MIAT or Ythdf2 group. $$$^p^ < 0.001 and ^$$^*p* < 0.01 and ^$^*p* < 0.05 vs siMIAT or siYthdf2 group. ns meat no significance. All data were presented as mean ± SD (*n* = 3).

Since MIAT and Ythdf2 were observed to be participants in hypertrophy and bound to each other in the above-mentioned results, we hypothesized that the pathological effect of MIAT in cardiac hypertrophy may be related to Ythdf2. To test this hypothesis, we transfected MIAT or Ythdf2 plasmid, siMIAT or siYthdf2 in H9c2 cells. In western blot analysis and RT-qPCR assays (Fig. 5A, B, E), the cells transfected with MIAT plasmid had increased mRNA and protein expression of cardiac hypertrophy markers ANP, BNP, and β-MHC, whereas PPARα and CPT-1a were notably decreased relative to mock or scramble groups. However, this tendency was rescued after co-transfection with siYthdf2. Similarly, knockdown of MIAT decreased cardiac hypertrophy markers ANP, BNP, and β-MHC, whereas PPARα and CPT-1a mRNA and protein levels were increased. However, this tendency was rescued after co-transfection with Ythdf2 plasmid. Consistent with these findings, MIAT overexpression had a significantly larger size than those mock or scramble groups. The hypertrophic cell surface area was mitigated when transfected with siYthdf2. Overexpression of Ythdf2 enlarged the cell surface area of siMIAT-transfected H9c2 cells (Fig. 5C, D). RT-qPCR assay showed that overexpression or knockdown of MIAT could influence the MIAT and Ythdf2 mRNA levels, whereas overexpression or knockdown of Ythdf2 could only influence Ythdf2 mRNA level (Fig. 5E). These results indicated that MIAT was the upstream of Ythdf2. MIAT regulated cardiac hypertrophy through Ythdf2.

Furthermore, the cells transfected with Ythdf2 plasmid had increased mRNA and protein expression of cardiac hypertrophy markers ANP, BNP, and β-MHC, whereas PPARα and CPT-1a were significantly decreased relative to control groups. However, this tendency was rescued after co-transfection with siMIAT. Similarly, knockdown of Ythdf2 decreased cardiac hypertrophy markers ANP, BNP, and β-MHC, whereas PPARα and CPT-1a mRNA and protein levels were increased. However, this tendency was rescued after co-transfection with MIAT plasmid in western blot analysis and RT-qPCR assays (Fig. 5F, G, J). These results were confirmed in phalloidin staining. Ythdf2 overexpression had a significantly larger size than those in control groups. The hypertrophic cell surface area was mitigated when transfected with siMIAT. Overexpression of MIAT enlarged the cell surface area of siYthdf2-transfected H9c2 cells (Fig. 5H, I). RT-qPCR confirmed that MIAT was the upstream of Ythdf2 (Fig. 5J). As anticipated, Ythdf2 functioned as a downstream of MIAT in cardiac hypertrophy.

### MIAT regulated cardiac hypertrophy via the m^6^A methylation reading protein Ythdf2 to regulate CPT-1a m^6^A modification

**Fig. 6:**
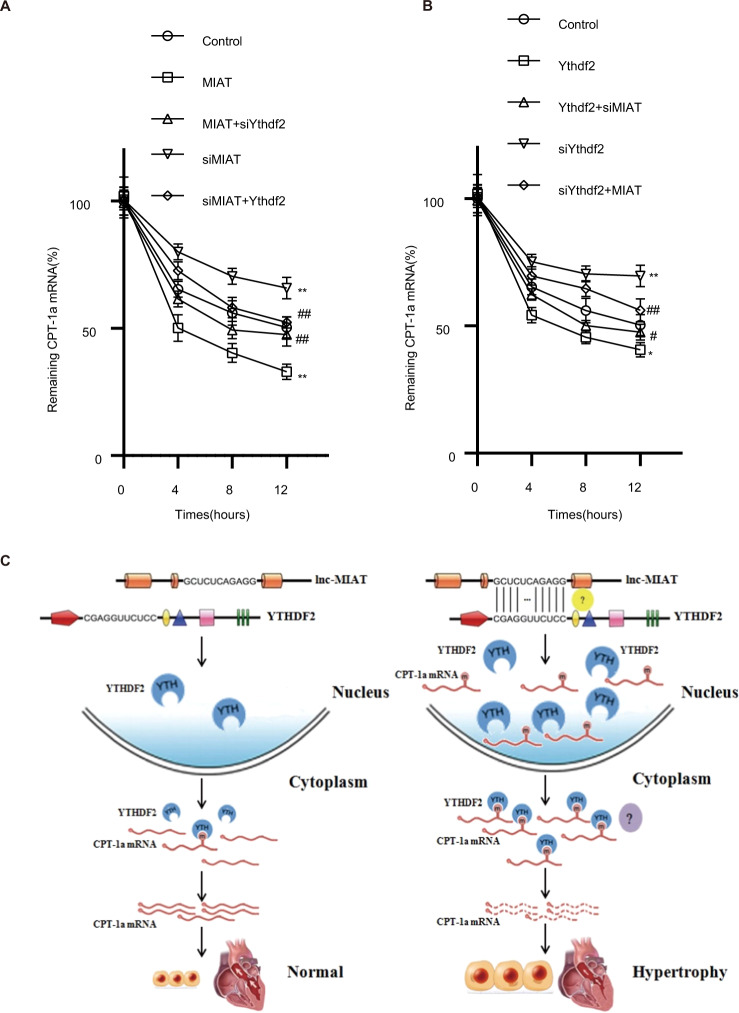
MIAT regulated cardiac hypertrophy via the m^6^A methylation reading protein Ythdf2 to regulate CPT-1a m^6^A modification. **A** RNA stability assay was performed in actinomycin D-treated H9c2 cells transfected with above plasmid or siRNA. mRNA of CPT-1a was detected by RT-qPCR at 0, 4, 8, and 12 h. **B** Schematic figure revealing the proposed signaling pathway linking MIAT, Ythdf2, CPT-1a to CH. **C** A proposed model showing MIAT regulates CPT-1a induced cardiac hypertrophy through Ythdf2.

We worked on the assumption that Ythdf2, as an m^6^A methylation reading protein, may bind to the m^6^A site of CPT-1a to promote CPT-1a mRNA degradation. The RNA stability assay (Fig. 6A, B) demonstrated that overexpression of MIAT would accelerate the degradation of CPT-1a mRNA after inhibition of transcription. Knockdown of Ythdf2 could reverse this trend in MIAT-transfected H9c2 cells. Contrary to the changes observed in the H9c2s after overexpression of MIAT, siMIAT would inhibit the degradation of CPT-1a mRNA, whereas for overexpression of Ythdf2 in siMIAT-transfected cells, the trend was rescued. Similarly, knockdown of MIAT in Ythdf2-transfected cells could enhance the CPT-1a mRNA stability, whereas overexpression of MIAT in siYthdf2-transfected cells would decrease the CPT-1a mRNA stability. These results demonstrated that MIAT regulated cardiac hypertrophy via the m^6^A methylation reading protein Ythdf2 to regulate CPT-1a m^6^A modification. It provided evidence for existence of a new signaling pathway leading to hypertrophic phenotypes: MIAT ↑ → Ythdf2 ↑ →PPARα/CPT-1a ↓ →cardiac hypertrophy (Fig. 6C).

## Discussion

In the current studies, we identified a lncRNA MIAT as a new hypertrophy-associated lncRNA, that promotes cardiac hypertrophic processes by regulating m^6^A RNA modification. Mechanistically, MIAT interacted with Ythdf2 and increased Ythdf2 expression. This effected PPARα/CPT-1a signaling and markedly suppressed the m^6^A modification of CPT-1a mRNA. However, CPT-1a mRNA level was downregulated and eventually led to CH. Taken together, the present study showed a new hypertrophic signaling pathway mediated by MIAT: MIAT/Ythdf2/PPARα/CPT-1a/CH (Fig. 6C).

The vital role of lncRNAs in CH has been studied over decades. For example, lncRNA Chrf binds to miR-489 and activates its target gene Myd88 in a cell and mouse of CH model [29]. LncRNA-ROR promotes CH via binding with miR-133 [30]. The first cardioprotective lncRNA Mhrt interacts with helicase domain of Brg1, which is a chromatin-remodeling factor, thus preventing Brg1 from identifying its genomic sequence and inhibits stress-induced CH and heart failure [31]. Herein, we demonstrated that MIAT was a hypertrophic lncRNA, and this lncRNA was markedly upregulated in CH induced by TAC in vivo or by AngII in vitro.

In our previous study, we demonstrated that downregulation of MIAT could inhibit the expression of cardiac hypertrophy indicators ANP and BNP in ISO-treated neonatal rat ventricular myocytes (NRVMs) [32]. Other studies reported similar results in diabetic myocardial changes [33], suggesting that MIAT was involved in the process of CH, but the detailed mechanism remained unclear. MIAT was discovered and named by Japanese scholar Nobuaki Ishii in 2006. It was first reported in human cells and then found in rats. Ishii first found that MIAT was significantly increased in peripheral blood of patients with myocardial infarction [34]. Vaughnsort discovered that MIAT could predict left ventricular dysfunction [35]. Recent studies reported that MIAT was associated with microvascular injury and myocardial fibrosis after infarction [36–38], suggesting that it involved in myocardial remodeling. MIAT had a high degree of sequence conservation in humans, rats, and mice [39], which made the study of MIAT in disease pathogenesis more practical.

Ythdf2 is one of the members of the methylation reading protein. It has been shown to selectively inhibit cancer stem cells in acute myeloid leukemia [40], modulate mice neural development [41], and maintain oncogene expression in glioblastoma stem cells [42]. In the cardiovascular field, Ythdf2 was increased in heart failure with preserved ejection fraction (HFpEF) patients, compared with healthy controls [43]. In heart failure mouse model constructed by transverse aortic constriction (TAC) surgery and primary cardiomyocytes stimulated with ISO model, researchers found that the mRNA and protein level of Ythdf2 was remarkably increased during HF development [44]. The above phenomenon was consistent with our study that Ythdf2 expression levels are upregulated in cardiac hypertrophy model in vivo and in vitro. Xu et al. showed that Ythdf2 overexpression could efficiently alleviate cardiac hypertrophy as a protective factor [44], whereas our experiments showed that Ythdf2 overexpression promoted cardiac hypertrophy of H9c2 cells. The difference may be due to modeling method. We used Ythdf2 plasmid in H9c2 cells while they used AAV9-Ythdf2 on TAC mice. In addition, we found that the level of Ythdf2 was upregulated by MIAT overexpression and downregulated by MIAT silencing. Such a positive correlation between Ythdf2 and MIAT expression suggests a targeting relationship. Our FISH results confirmed this hypothesis: MIAT was located in cell nucleus and cytoplasm, while Ythdf2 was located in cytoplasm. A number of lncRNAs have been reported to regulate gene by cis regulation or trans regulation according to the distance. In our case, it is likely that the mechanism is trans regulation.

Peroxisome proliferator-activated receptor α (PPARα) signaling was involved in cardiac hypertrophy. For example, in AngII-induced hypertrophic H9c2 cells, luteolin could improve abnormal glucolipid metabolism by inhibiting the expression of HIF-1α and then modulating PPARα signaling, activating its target genes, including CPT-1a, PDK-4, and GLUT-4 [27]. Osthol could reduce rat cardiac hypertrophy by increasing PPARα mediated CPT-1a mRNA while decreasing DGAT mRNA [28]. Another study revealed that transfecting Ythdf2 plasmid into liver cells and then enrichment of mRNA by antibody, resulted in Ythdf2 binding to PPARα gene and affecting its stability and gene expression [45]. This study showed that Ythdf2-regulated PPARα in liver cells. Combined with bioinformatics analysis, we speculated that MIAT, Ythdf2, and CPT-1a have an interaction relationship. Our experimental results supported the rationality and feasibility of the hypothesis.

Perhaps the most remarkable finding of our research was the demonstration that MIAT plays an important regulatory role in cardiac hypertrophy through the m^6^A methylated reading protein Ythdf2 in vitro cellular model. We found that MIAT and Ythdf2 were significantly increased during cardiac hypertrophy. This project will provide a new idea and direction for the clinical study of cardiac hypertrophy prevention and treatment strategies, which is important for elucidating that the mechanism of cardiac hypertrophy and has scientific significance.

However, our study has some limitations: First, the role of MIAT and Ythdf2 in m^6^A RNA methylation modification is limited, and more experiments need to be implemented to explain this phenomenon. Second, total and CPT-1a m^6^A methylation levels in H9c2 cells need to be measured in our future study. Third, the effect of m^6^A motif point mutation on cardiac hypertrophy need to be studied further.

Epitranscriptome based on drugs or other methods can ameliorate the hypertrophic heart. By interfering with the expression level of m^6^A methylase with various techniques, for example, overexpress METTL3 with adenovirus, transfect METTL3 siRNA, and use METTL3 and FTO knockout mice, etc., researchers found that the cardiac m^6^A RNA modifications could regulate gene expression and influenced cardiac hypertrophy [13]. These results suggest that RNA methylation may affect mRNA post-transcription level and downstream translation. RNA-directed therapy is widely accepted and routinely used to study cardiac pathophysiology.

It should be noted that in our model, increased expression level of MIAT promoted hypertrophic responses of the heart (MIAT ↑ → Ythdf2 ↑ →PPARα/CPT-1a ↓ →CH); upregulation of m^6^A RNA methylation reading protein Ythdf2 resulted in CPT-1a mRNA expression level decrease. This would indicate a harmful action of MIAT.

## Supplementary information

Supplemental figure 1

Original full length western blot-1

Original full length western blot-2

## Data Availability

The data used to support the findings of this study are included within the article.
