# Supplementary figures and images for: The lncRNA MIAT regulates CPT-1a mediated cardiac hypertrophy through m6A RNA methylation reading protein Ythdf2
Source: Cell Death Discov. 2022 Apr 5;8:167. doi: 10.1038/s41420-022-00977-8 (PMC8983679; doi:10.1038/s41420-022-00977-8)

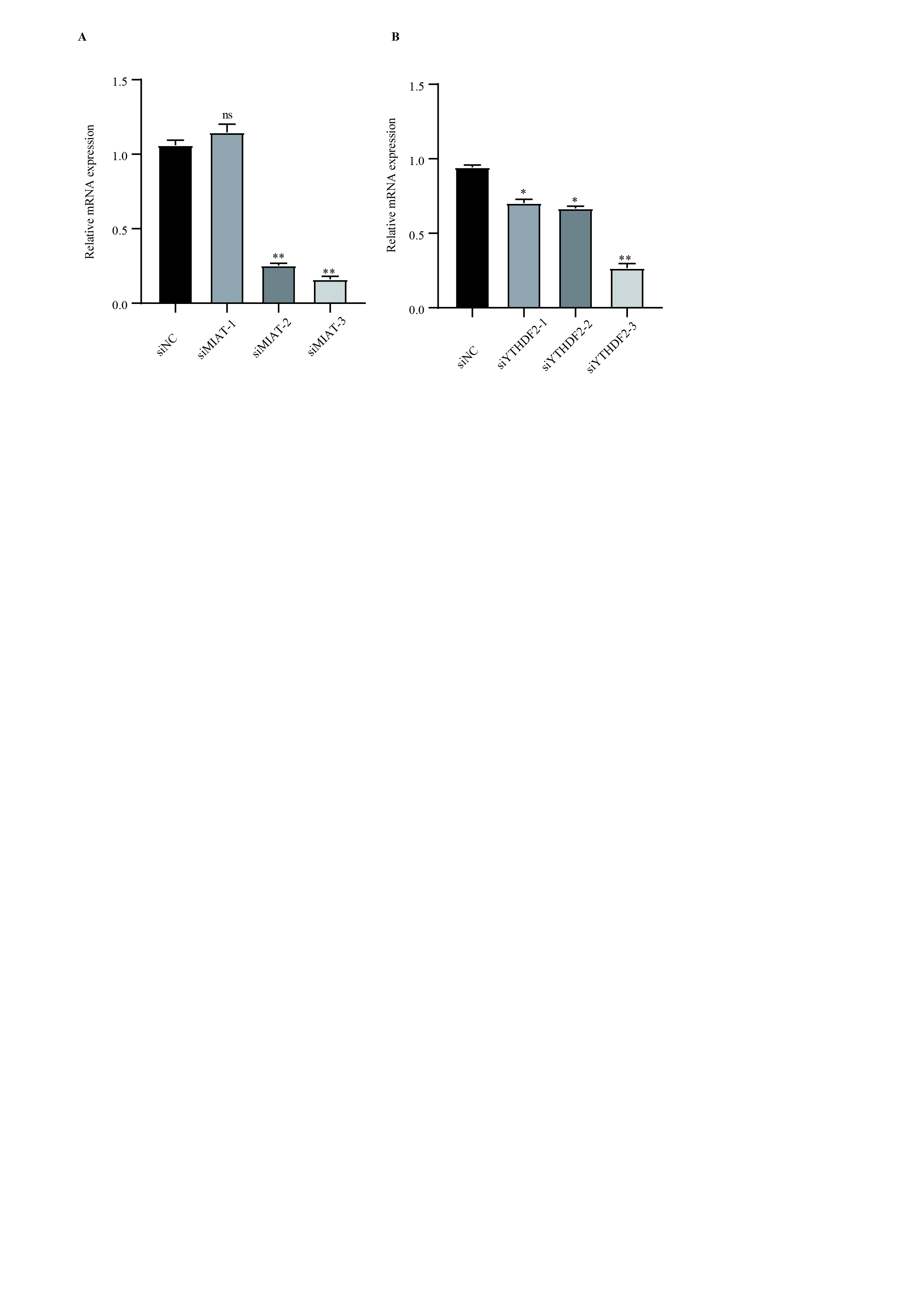

Supplement: Supplementary file 1 — Supplemental figure 1 [file 41420_2022_977_MOESM1_ESM.jpg]

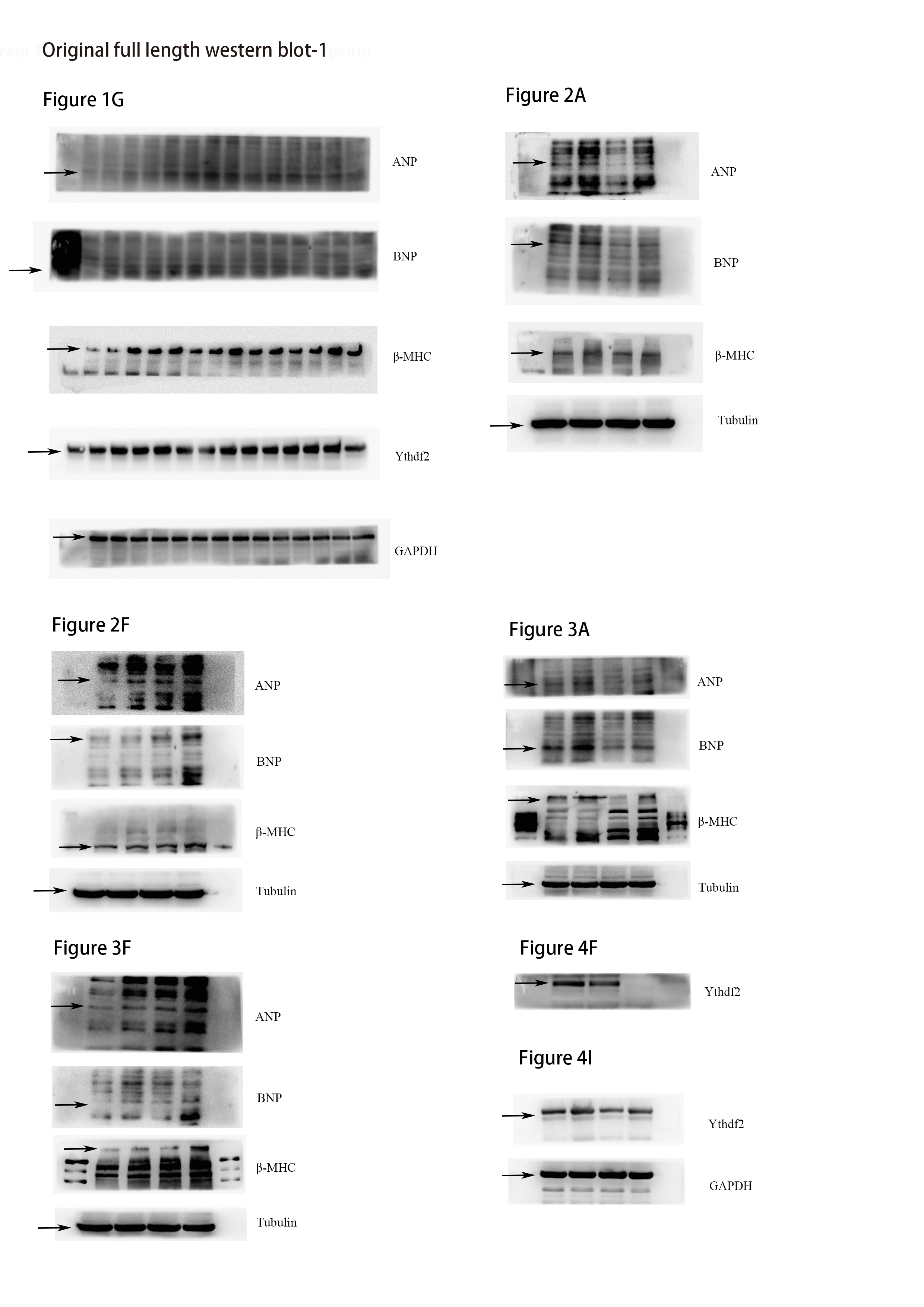

Supplement: Supplementary file 2 — Original full length western blot-1 [file 41420_2022_977_MOESM2_ESM.jpg]

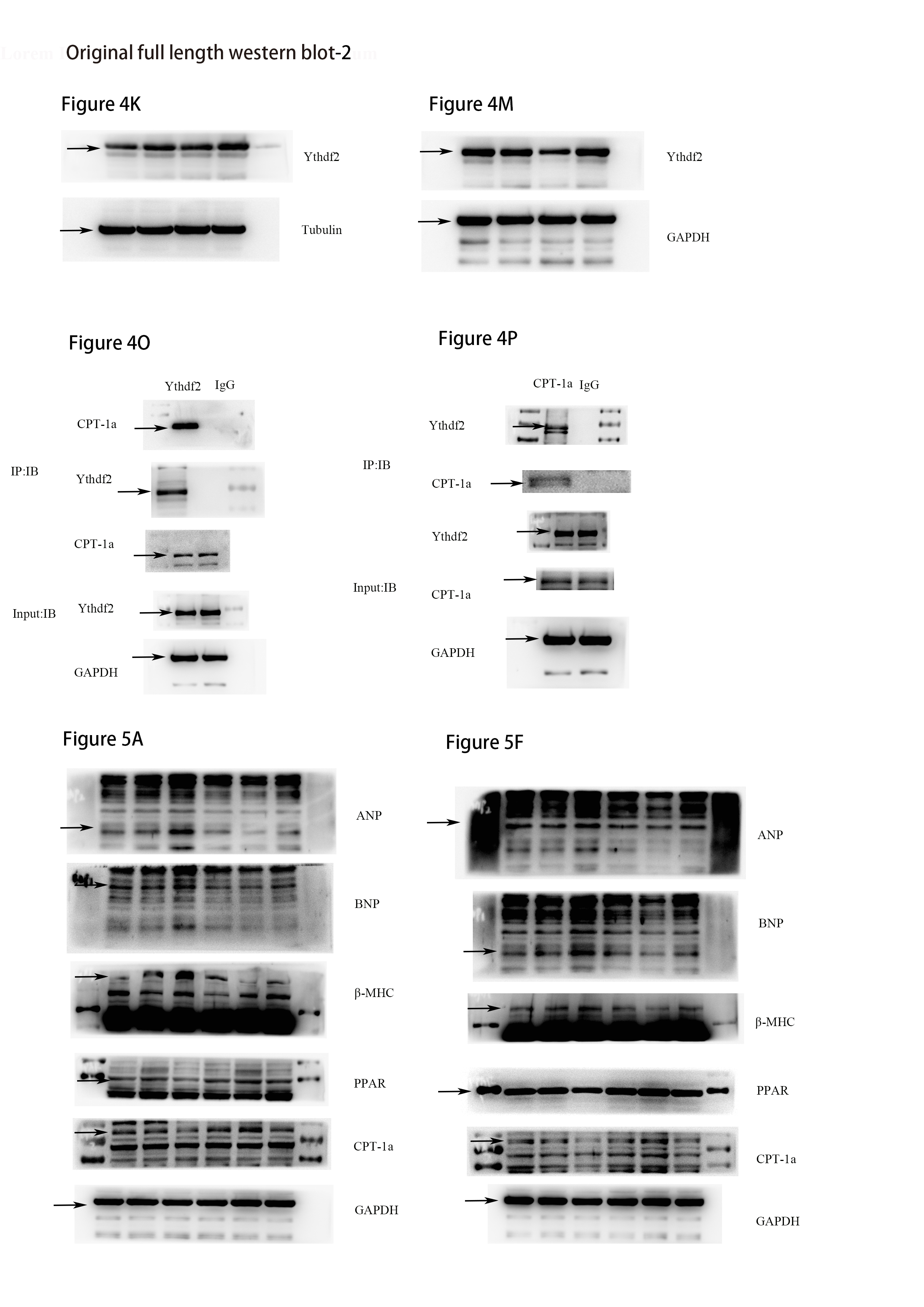

Supplement: Supplementary file 3 — Original full length western blot-2 [file 41420_2022_977_MOESM3_ESM.jpg]
